# The positive effects of time spent in nature on stress: considering climate change

**DOI:** 10.1038/s41380-023-02122-y

**Published:** 2023-06-13

**Authors:** Sebastian Ocklenburg

**Affiliations:** 1 Department of Psychology, MSH Medical School Hamburg, Hamburg, Germany; 2 ICAN Institute for Cognitive and Affective Neuroscience, MSH Medical School Hamburg, Hamburg, Germany; 3 Biopsychology, Institute of Cognitive Neuroscience, Faculty of Psychology, Ruhr University Bochum, Bochum, Germany

**Keywords:** Psychiatric disorders, Neuroscience

## To the Editor:

Building upon previous work demonstrating that the prevalence of psychiatric disorders rises with increasing urbanization [1], Sudimac et al. demonstrated in *Molecular Psychiatry* that spending time in a natural environment can reduce activity in the amygdala, an important key structure in stress-related brain networks [2]. The authors compared brain activity during a social stress test after a walk in a forest to brain activity after a walk on a busy street. They found that walking in nature decreased amygdala activity, while it stayed the same after a walk in an urban environment. Since stress is a major risk factor for several psychiatric disorders, these findings suggest that walking in nature may have protective or salutogenic effects on the pathogenesis of psychiatric disorders. The authors conclude that urban planners should consider this evidence and ensure to include enough accessible green space in cities. While I do agree with these conclusions, I would like to point out that the finding that spending time in nature can downregulate stress, a major risk factor for psychiatric diseases, has more wide-reaching implications than are discussed in the article, especially in the context of the ongoing climate crisis.

Anthropogenic climate change is one of humanity’s greatest challenges in the twenty-first century and has major effects on natural spaces around the world. Importantly, it has been shown that the effects of climate change, like rising temperatures, a higher probability of natural disasters, and reduced access to food and water resources, have detrimental effects on cognitive function and mental health, and lead to an increase in the prevalence of psychiatric disorders [3]. The climate crisis thus is likely to become as much of a mental health crisis as an ecological crisis. For example, while pleasant warm weather has been linked to a more positive mood and better memories [4], heat waves with uncharacteristically higher temperatures have been linked to decreased cognitive functions [5]. Moreover, higher temperatures have been linked to an increase in interpersonal violence and intergroup conflict [6, 7]. In addition, anthropogenic climate change has been linked to an increased prevalence of events that adversely affect mental health. For example, California experienced a fivefold increase in the annual burned area due to wildfires during 1972–2018 [8]. The experience of wildfires has been linked to psychosomatic responses, anxiety, depression, insomnia, and other adverse effects [9]. As an increased number of wildfires is also more likely to affect more and more areas containing human settlements, an increase in such adverse effects on mental health is to be expected. In addition, wildfires may cause air pollution in surrounding areas which has been linked to increased risk of depression symptoms, as well as to changes in brain structure such as decreased gray-matter volume in brain networks connecting the cortex to the striatum and the thalamus [10]. Furthermore, anthropogenic climate change has been linked to an increase in flood size [11], and catastrophic flood events in turn result in an increase in post-traumatic stress disorder, anxiety, and depression [12]. Moreover, climate change has been identified as leading to an increase in risk factors for various neurological brain disorders. For example, it has been suggested that changing climate conditions may lead to changes in the transmission of brain-infecting pathogens or parasites and pollution of the environment associated with climate change may be associated with an increased exposure to neurotoxicants [13]. In addition to these direct pathogenic effects of exposure to events caused by climate change on mental health, also more indirect effects of climate change on mental health have been observed. For example, it has been reported that an increasing number of people worldwide experience climate-change-related anxiety issues [14]. Taken together, anthropogenic climate change leads to an increase in pathogenic factors for several psychiatric disorders such as post-traumatic stress disorder, anxiety, and depression. Thus, it will likely result in a long-term increase in the prevalence of such disorders. The results by Sudimac et al. [2], however, also suggest that the negative effects of anthropogenic climate change on mental health do not end there. As natural disasters like wildfires and floods destroy large quantities of natural space, climate change also leads to a reduction in the availability and accessibility of such spaces. Moreover, climate change has been shown to lead to a loss of biodiversity in natural spaces [15]. This may reduce the salutogenic effects of such spaces that have been demonstrated by Sudimac et al. [2]. Therefore, climate change does not only lead to an increase of pathogenic factors for many psychiatric disorders but a parallel decrease in the availability of an important salutogenic factor. Thus, the results by Sudimac et al. [2] suggest that the mental health toll of anthropogenic climate change may be even worse than predicted on the increase of pathogenic factors for psychiatric disorders alone. Therefore, it will be crucial to systematically investigate the pathopsychology of climate change over the next decade, focusing on multidisciplinary collaborations between psychiatrists, neuroscientists, climate scientists, neurologists, and clinical psychologists. The experimental fMRI paradigm introduced by Sudimac et al. [2] provides an excellent conceptual blueprint for future research aimed at examining the effects of climate change on brain functioning and mental health. For example, it could be measured how the brain’s stress response is modulated by walking through an intact forest with living trees and high biodiversity compared to walking through a forest that has been severely damaged by the consequences of climate change such as drought or forest fires. This variation of the paradigm would give an ecologically valid way to measure how the climate crisis modulates how individuals process stress, which in turn is highly relevant for mental health. It could be adapted to all types of natural environments, not only forests. As several environmental consequences of the climate crisis make environments entirely unhospitable, such as forests during or after a forest fire, a real-life walk in the respective environment may not always be possible. If safety reasons prohibit walking through a specific environment, the paradigm introduced by Sudimac et al. [2] could also be combined with highly immersive virtual reality scenarios of such environments. To this end, head-mounted virtual reality displays and walking treadmills could be used to measure the effects of impactful events such as forest fires or floods on the stress response without compromising participants’ safety. Finally, these variations of the paradigm could also be used in groups of patients suffering from climate-change-related mental health issues such as climate anxiety. Comparing the environment-driven modulation of the stress response between such groups and unaffected individuals could yield highly relevant insights into the mental health effects of the climate crisis.
